# The Emulsifying Properties of Hydrogenated Rosin Xylitol Ester as a Biomass Surfactant for Food: Effect of pH and Salts

**DOI:** 10.3390/molecules25020302

**Published:** 2020-01-12

**Authors:** Hong Qiu, Xiaopeng Chen, Xiaojie Wei, Jiezhen Liang, Dan Zhou, Linlin Wang

**Affiliations:** 1 School of Chemistry and Chemical Engineering, Guangxi University, Nanning 530004, China; qiuhong@mail.gxu.cn (H.Q.); lilm@gxu.edu.cn (X.C.); wxjgxu@gxu.edu.cn (X.W.); ljztonyol@163.com (J.L.); 1814404054@st.gxu.edu.cn (D.Z.); 2 Guangxi Key Laboratory of Petrochemical Resources Processing and Process Intensification Technology, Guangxi University, Nanning 53004, China

**Keywords:** xylitol ester of hydrogenated rosin, emulsifying properties, water-in-oil emulsions, pH, salts

## Abstract

The xylitol ester of hydrogenated rosin (XEHR) was obtained for the first time from biomass-based hydrogenated rosin and xylitol using an environmentally friendly, high-pressure CO_2_ catalytic synthesis. This compound is intended for use as an emulsifier for food. Analyses by ICP-AES showed the absence of heavy metal residues in the product, such that it met food standards. Fourier transform infrared and nuclear magnetic resonance spectroscopies together with gel permeation chromatography confirmed the successful esterification and the formation of a monoester and diester with molar masses of 427 and 772 g/mol. The emulsification of water/soybean oil mixtures by adding the XEHR was assessed at pH values of 4, 6.86, and 10 and in the presence of NaCl, KCl, MgCl_2_, and CaCl_2_. The XEHR was found to act as an emulsifier by reducing the interfacial tension of such mixtures to less than 2 mN/m under all conditions. The highest emulsifying activity index (9.52 m^2^/g) and emulsifying stability index (94.53%) were obtained after adding MgCl_2_ (100 mM). Particle size and confocal microscopy showed that the presence of salts gave a more uniform droplet size and a finer emulsion structure. The high viscosities of the emulsions containing salts also suggested a more cohesive oil droplet network.

## 1. Introduction

An emulsifier is a type of surfactant capable of rapidly reducing the interfacial tension between oil and water [1]. These compounds are important ingredients in forming stable emulsions with suitable shelf-lives and functional properties [2], and they are widely used in foods [3], cosmetics [4] and catalysis [5], and as templates for the preparation of advanced materials [6]. Emulsifiers employed in the food industry come from a wide range of sources, including milk (or whey), eggs, soybeans, soluble polysaccharides, pectin, and gum arabic [7]. The emulsifying properties of such materials are attributed to the specific carbohydrates or proteins that they contain [8]. At present, there are few commercial applications for artificial emulsifiers because these are synthesized using toxic solvents and thus can contain residues of such toxins [9]. In contrast, emulsifiers modified by the addition of proteins are widely used because of their high nutritional value [10] and excellent functional characteristics [11]. However, some studies have shown that these proteins may act as common food allergens. There are also rising concerns related to dietary restrictions associated with milk and egg proteins, the spread of diseases such as bovine spongiform encephalitis, and multidrug-resistant food-borne pathogens [12]. In addition, because these emulsifiers exhibit different properties depending on the acidity/basicity or salt content of their environment [13], it is challenging to expand their applications.

The manufacturers of emulsion products must understand the behavior of these surfactants under different conditions, such as over a wide pH range or in the presence of salts, so as to select the most suitable surfactants for specific applications. For all these reasons, it would be of interest to develop environmentally friendly methods of synthesizing stable emulsifiers and to study the emulsion behavior of these compounds under different conditions.

Rosin is a nonvolatile solid obtained from living pine trees and comprises a mixture of resin acids having different molecular structures [14]. As a natural renewable resource, rosin is both inexpensive and abundant, with an annual production worldwide of approximately 1.2 million tons [15]. Hydrogenated rosin is one of the main products obtained from this material because it has the advantages of a light color, oxidation resistance, and minimal brittleness [16]. In addition, the conjugated double bonds in rosin can be hydrogenated while retaining the hydrophobic nature of the tricyclic phenanthrene skeleton and the carboxyl groups. Thus, hydrogenated rosin can be used as a raw material for the synthesis of surfactants [17,18]. In recent years, biomass-based food additives have been synthesized from hydrogenated rosin because this represents a “green” raw material [19]. As an example, the well-known hydrogenated rosin glyceride, which can be used as an emulsifier and density modifier in nonalcoholic drinks and turbid drinks, has been listed as a food additive in the United States, Japan, Spain, and China [20,21]. However, the carboxyl group in hydrogenated rosin is connected with the tertiary carbon atom, having a strong steric effect, which makes the reaction activation energy of the carboxyl group high. Thus, research concerning the hydrogenated rosin esterification process of hydrogenated rosin not only focuses on improving technical performance but also on the design of clean synthetic routes. As a means of providing an environmentally friendly reaction medium for chemical synthesis, high-pressure CO_2_ can be used to replace traditional solvents [22]. In recent years, many research studies have shown that pressurized CO_2_ can be used as a “green” reaction medium, in particular for catalysis.

Xylitol, a five-carbon sugar alcohol, is a naturally occurring polyalcohol [23]. Because this compound is highly water soluble, it has been widely used in various food products, such as sugar-free gum and baking products [24]. Xylitol does not need insulin to participate in the metabolism of the body, and it does not increase the blood sugar value; therefore, the nutritive sweetener xylitol is being considered as a possible alternative for glucose and sucrose in the diet of diabetic patients [25]. At the same time, xylitol has some special physiological functions such as preventing caries, eliminating blood ketone disease, and improving liver function [26]. Xylitol fatty acid esters can also act as surfactants [27]. Moreover, sugar-based fatty acid esters are commercially used as emulsifiers in foods at concentrations as high as 10 mg/mL, which reflects their low toxicities. The maximum daily intake for these materials is also quite high at 20 mg/kg body weight [28].

**Figure 1:**
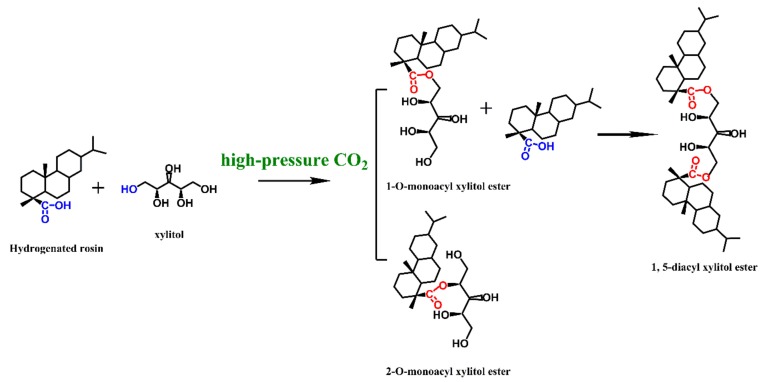
Reaction network diagram of hydrogenated rosin and xylitol.

In this work, the xylitol ester of hydrogenated rosin (XEHR) was synthesized for the first time, using xylitol as the hydrophilic portion of the molecule and hydrogenated rosin as the hydrophobic portion, in conjunction with high-pressure CO_2_ (Figure 1). The objectives of this investigation were to characterize the chemical structure and physical properties of XEHR as a chemically synthesized emulsifier, as well as to determine the surface activity of this molecule and the properties of soybean oil/water/XEHR emulsions. The emulsions were evaluated while varying both pH and salt type, and rheometry, particle size analysis, and microscopy were used to explore the relationship between the emulsion microstructure and the emulsifying properties of the XEHR. The raw materials used in this experiment were obtained from biomass (a renewable feedstock), the synthetic method was environmentally friendly, and the product was found to have a stable chemical structure. It is expected that XEHR may have a number of applications in acidic, basic, or high-salt emulsion systems.

## 2. Results and Discussion

### 2.1. Preparation

#### 2.1.1. Conversion Values

**Figure 2:**
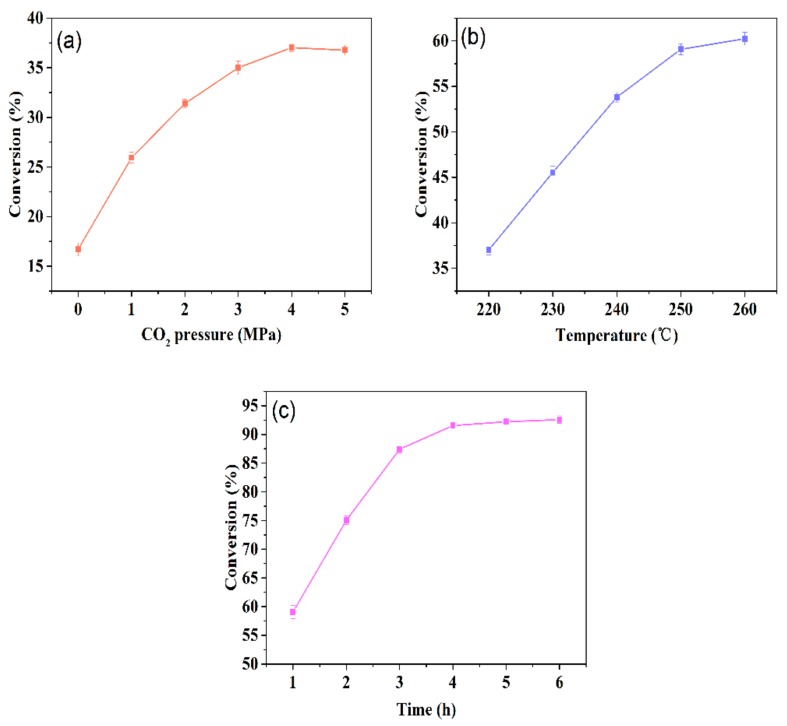
Hydrogenated rosin conversion versus various reaction conditions. Reaction conditions: stirring speed, 400 r/min; the molar ratio of hydrogenated rosin to xylitol, 1:1. (**a**) Temperature, 220 °C; time, 1 h. (**b**) Pressure of CO_2_, 4 PMa; time, 1 h. (**c**) Pressure of CO_2_, 4 PMa; temperature, 250 °C.

In this work, esterification was carried out under various conditions, and the relevant results are shown in Figure 2. The initial conditions of this experiment were as follows: temperature, 220 °C; reaction time, 1 h. As there is no relevant report on the production of rosin xylitol ester, according to the existing ASTM D-465 standard [29], the conversion value of rosin ester must be greater than 90%. Without any catalyst, the esterification rate was only 16.70% after one hour. When 1 MPa CO_2_ was filled into the reaction system, the esterification rate rose to 25.95% after one hour. When the CO_2_ pressure in the system rose from 1 to 4 MPa, the conversion rate increased from 25.95% to 37.02%, but when the CO_2_ pressure changed from 4 to 5 MPa, the conversion rate did not change significantly. It shows that 4 MPa CO_2_ has reached the catalytic saturation. Considering the cost, the following experiments were carried out at 4 MPa. At the same time, because significant activation energy is needed for esterification, it is very important to choose the reaction temperature. When the temperature increased from 220 to 250 °C, the conversion increased from 37.02% to 59.07%. When the reaction temperature increased from 250 to 260 °C, the conversion increase was very limited, and the color of the product deepened. First of all, the activation energy of esterification can be reached by increasing the temperature, but the pH of the reaction system will also increase with the increase of temperature, which reduces the catalytic effect of CO_2_, and the resin acid will cause cracking at high temperature. Because there are multiple competing reactions in the system, the reaction temperature was determined as 250 °C. Next, the effect of reaction time was studied. When the reaction time was extended from 1 to 4 h, the conversion was over 90%. As the esterification reaction is reversible and has a reaction limit, the effect of prolonging the reaction time on the conversion rate was not obvious after 4 h. Finally, the best reaction conditions were determined: temperature, 250 °C; CO_2_ pressure, 4 MPa; time, 4 h.

Finally, the esterification experiments were carried out under three conditions with the optimal reaction conditions above: (1) with no catalyst, (2) with the traditional ZnO catalyst (0.8%), and (3) with high-pressure CO_2_ as the catalyst (4 MPa), and the associated esterification rates were ascertained. As shown in Table 1, only partial esterification was obtained without the catalyst, and the conversion rate of the hydrogenated rosin was unsatisfactory. Using the ZnO, the conversion percentage was increased to 78.52%, but the required conversion rate of 90% within 4 h was not obtained. In contrast, 91.51% conversion was obtained after 4 h with the high-pressure CO_2_ catalysis. These data confirm that high-pressure CO_2_ exhibits superior catalytic activity during esterification.

#### 2.1.2. Hydrophilic–Lipophilic Balance (HLB) Value

**Figure 3:**
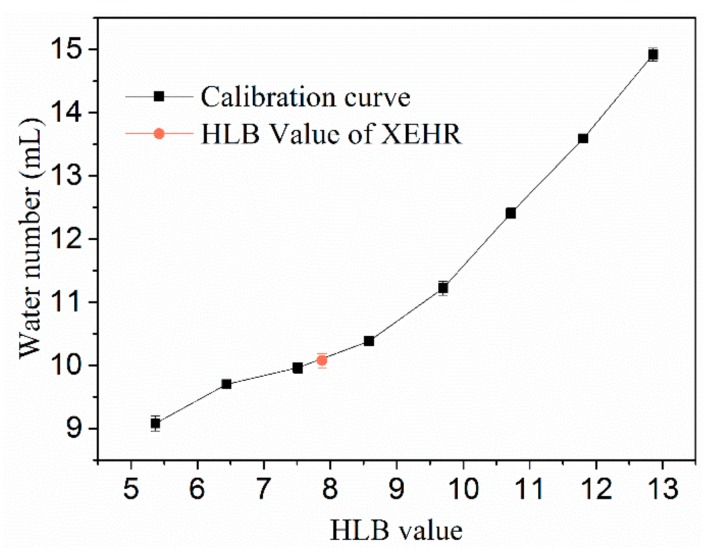
The hydrophilic–lipophilic balance (HLB) calibration curve (based on Greenwald et al.) and the HLB values for the XEHR. The plot of water number vs. HLB was obtained from Equation (2) using span-80 and tween-80 mixtures.

Emulsifiers having low HLB values tend to be oil soluble, and materials having high values tend to be water soluble. Calculation of HLB values of surfactant is very important in product quality and yield points of view [30]. There are many kinds of rosin derivatives as surfactants [31], which have different applications according to their HLB values. For example, rosin glyceride mentioned in the introduction, will generally be mixed with other surfactants or used as emulsifier after further modification (such as the reported rosin-based polymer, prepared by the establishment of rosin with glycol and pentaerythritol [32]). When pentaerythritol was mixed additionally, the HLB value can reach 9.92, which can be used as a cream base. The reported rosin imides maleic anhydride adduct is a surfactant that can be used in organic solvents [33]. A kind of polymer of coconut oil, rosin, and maleic anhydrides, whose HLB value can reach 13.82, can be used in making liquid, powder, and cake detergent [34]. The HLB values determined for the XEHR are presented in Figure 3. The average HLB value for the three groups of samples was 7.81, which falls within the range of suitable values for the preparation of oil in water emulsions.

#### 2.1.3. ICP-AES

Compared with the product synthesized using ZnO, the Zn concentration in the product obtained using high-pressure CO_2_ was much lower, and it was approximately equal to that in the hydrogenated rosin (Table 2). The Zn in the hydrogenated rosin and the increase in the trace Zn level in the sample after the reaction are attributed to contamination from the raw material packaging and residues from the reactor. The Cu, Fe, Ni, and Pb concentrations in the XEHR met the requirements for use of this material as a food additive according to U.S. Food and Drug Administration standards [29].

### 2.2. Characterization

#### 2.2.1. FTIR

FT-IR spectra of the XEHR and hydrogenated rosin can be found in the Supplementary Materials (see Figure S1). In the spectrum obtained from the hydrogenated rosin, the peak at 1276 cm^−1^ is attributed to the stretching vibration of the carboxylic acid C-O bond, while that at 1695 cm^−1^ results from the stretching vibration of the C=O bond in the same moiety. Following the reaction with xylitol, the peak associated with the C-O bond is shifted to 1242 cm^−1^, indicating the C-O-C stretching vibration of an ester group. In addition, the C=O peak is moved to 1727 cm^−1^, confirming that the hydrogenated rosin was converted to XEHR. The intense, broad peak at 3443 cm^−1^ is characteristic of the stretching vibration of the O-H bonds in xylitol. These results demonstrate a higher degree of esterification in the product [35].

#### 2.2.2. NMR

^1^H and ^13^C NMR spectra can be found in the Supplementary Materials (see Figure S1). Here, a chemical shift, δ (^13^C), of 178.51 ppm is ascribed to the ester group, while the peaks at 4.01, 4.04, and 3.74 ppm (^1^H) and 65.44, 65.44, and 69.46 ppm (^13^C) are ascribed to the 1-O-monoacyl xylitol ester. The small peaks at 4.88 ppm (^1^H) and 75.26 ppm (^13^C) are due to the H2/C2 atoms of the 2-*O*-monoacyl xylitol ester. In addition, the peaks at 3.73 ppm (^1^H) and 68.88 ppm (^13^C) correspond to the methyl group in the 1,5-diacyl xylitol ester. Therefore, the hydrogenated rosin and xylitol evidently formed two esters in the reaction process. No evidence for a triacyl xylitol ester was found, which may partly be due to steric hindrance [36,37,38].

#### 2.2.3. GPC

**Figure 4:**
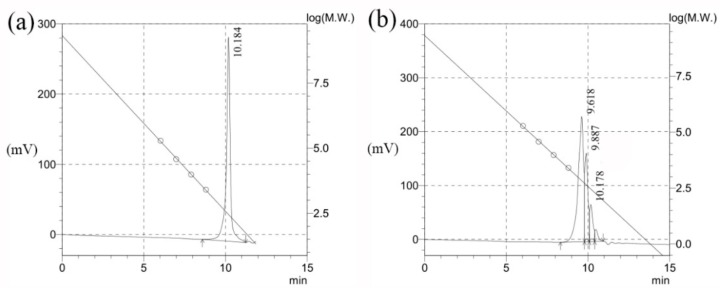
GPC chromatograms and calibration curves for (**a**) the hydrogenated rosin and (**b**) the XEHR.

The GPC data obtained from analysis of the raw hydrogenated rosin and of the reaction mixture after 4 h are provided in Figure 4, while the molecular weights and molecular weight distributions are summarized in Table 3. These results verify the presence of mono- and diesters in the samples. The raw rosin produced a single peak at a retention time of 10.184 min, equivalent to an Mr of 276, demonstrating that the raw material contained only the resin acid. After 4 h of reaction, two peaks appeared at 9.618 and 9.887 min, corresponding to Mr values of 772 and 427, respectively. These are consistent with the respective molecular weights of the diacyl and monoacyl xylitol esters and, thus, are consistent with the NMR results. Notably, the peak associated with the diacyl xylitol ester was the largest. Thus, during the 4 h reaction, the rosin not only reacted with the xylitol to form a significant quantity of the monoester, but it also continued to react to produce the diester. The polydispersity index for each of these peaks was close to 1, indicating that there were almost no side reactions.

### 2.3. Emulsification Properties

#### 2.3.1. Surface and Interfacial Tensions

**Figure 5:**
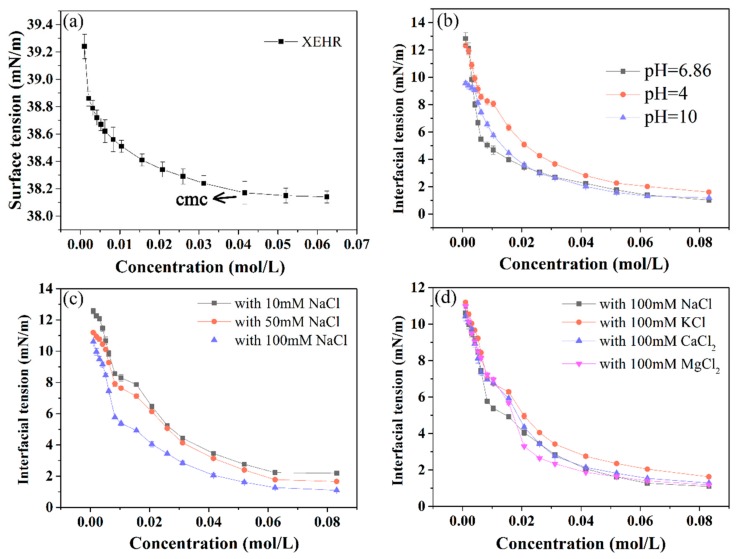
The surface tension curves for (**a**) XEHR in pure soybean oil, and for XEHR in oil/soybean mixtures showing the effects of (**b**) pH, (**c**) salt concentration, and (**d**) salt type.

The interfacial tension at a liquid–liquid interface is a crucial factor related to the study and characterization of emulsion stability. As an example, the introduction of surfactant molecules prevents the coalescence of oil droplets by reducing the interfacial tension of the mixture [39]. The surface tension curve for soybean oil containing XEHR is shown in Figure 5a, and the critical micelle concentration (CMC) value can be determined from the inflection point of this plot. It can be seen from these data that the XEHR had only a limited effect in terms of reducing the surface tension of pure soybean oil. This is attributed to the lower surface tension of the soybean oil itself and the irregular arrangement of surfactant molecules on the surface of the oil [40]. However, when water is added, the hydrophilic head of the XEHR molecule positions itself in the aqueous phase while the hydrophobic tail extends into the oil phase, such that the interfacial tension between the soybean oil and water is greatly reduced. Figure 5b–d illustrates the effects of pH, salt concentration, and salt type on the interfacial tension. It can be seen from the figure that, in general, XEHR had a good ability to reduce the interfacial tension in different pH and salt environments. However, the ability of XEHR to reduce the interfacial tension under acidic conditions was slightly weaker than that in neutral and alkaline environments. The reason is that different pH values may lead to changeable intra- and intermolecular repulsions of XEHR, which neutralizes the surface charge of XEHR and reduces the surface potential of particles [41]. Because the molecular weight of the hydrophobic group in XEHR is larger, XEHR is more lipophilic, especially when the charge is almost lost, and it is easy to be trapped in the oil [8]. However, since XEHR is not a polyelectrolyte, the effect of pH on its interfacial properties is finite. As shown in Figure 5c, when 10 mm NaCl is contained in the aqueous solution, the interfacial tension slightly increased at the same concentration of XEHR, compared with that in the neutral environment, but with the increase of salt concentration, XEHR gradually showed a better ability to reduce the interfacial tension. According to the report, the presence of cations (e.g., Ca^2+^ and Na^+^) may shield the negative charges of surfactant movements, thus decreasing the repulsive force between drops [40]. At the same time, NaCl was beneficial to promote surface movements to transfer from the bulk solution to oil–water interface, compress the electrical double layer, and form a compact surfactant molecule arrangement on the interfacial layer [42]. Because of the interaction of the two effects, the interfacial tension increased first and then decreased with the increase of salt concentration. In other kinds of salt solutions, it also showed low interfacial tension (Figure 5d), especially in NaCl and MgCl_2_. Under these conditions, the surface tension was decreased below 2 mN/m, and the interfacial properties were better than those reportedly obtained from other artificial emulsifiers [6]. These results confirm that the XEHR is capable of forming effective emulsions.

#### 2.3.2. Emulsifying Activity

The emulsifying activity index (EAI) is an indicator of formation ability of an emulsion, while the emulsifying stability index (ESI) reflects the ability of an emulsion to maintain stability. Table 4 shows the emulsification performance of the XEHR under different conditions. The emulsifying activity without the XEHR was low, such that the emulsion was uneven and relatively unstable. The EAI values of the XEHR emulsions were also found to increase in neutral or alkaline environments. The lowest EAI of 7.11 m^2^/g appeared at a pH of 4, with an ESI of 75.56, showing that the emulsion stability was poor in acidic environments. In contrast to the majority of modified protein surfactants, the XEHR emulsions containing high salt concentrations exhibited higher EAI values, and the ESI also increased with increases in the NaCl concentration. The highest EAI appeared in the emulsions containing 100 mM NaCl or MgCl_2_, while the stabilities of emulsions containing MgCl_2_ and KCl were slightly higher than those for emulsions made with NaCl or CaCl_2_. These data are in good agreement with previous reports of the effects of inorganic salts such as NaCl, KCl, and CaCl_2_ on emulsion stability, and they demonstrate that the stability of some emulsions increase with increases in salt concentration [43]. The reason may be that, after the emulsion formed by XEHR, the droplets in the oil phase are equivalent to many micro interfaces. The surface charge of the droplet is not zero, and the droplets are kept stable by electrostatic exclusion. Just as electrostatic interaction can neutralize interfacial charges and make XEHR far away from the interface, the change of pH also weakens the electrostatic repulsion between droplets, resulting in flocculation and in low stability of emulsion in acidic environments [44], but this effect is not significant. Similarly, as salt affects the interfacial tension of soybean oil/water with XEHR, the stability of emulsion in salty environments also showed the same rule as that of interfacial tension tests. Overall, the XEHR showed a similar emulsifying activity to that reported for protein-based emulsifiers [45], which indicates that XEHR has the potential to replace protein-based emulsifiers in the food industry [46]. The emulsification behavior primarily depends on the stability of the surfactant structure and molecular flexibility [47]. Thus, bio-based surfactants that are highly readily adsorbed at the oil–water interface will produce better emulsification activity and stability.

#### 2.3.3. Microstructures of Emulsions

**Figure 6:**
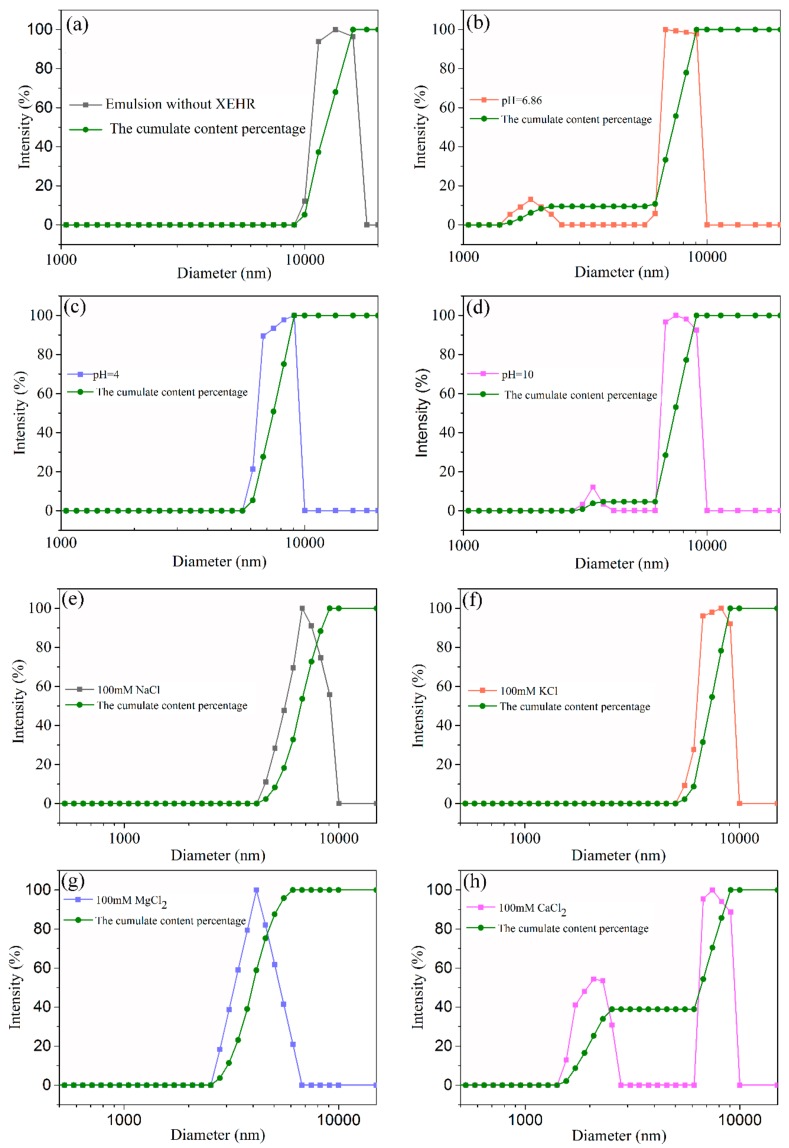
The particle size distribution curves and cumulative content percentage curves of emulsions under different pH and different salt conditions. (**a**) Emulsion without the XEHR, (**b**) emulsion pH = 6.86, (**c**) emulsion pH = 4, (**d**) emulsion pH = 10, (**e**) emulsion containing NaCl 100 mM, (**f**) emulsion containing KCl 100 mM, (**g**) emulsion containing MgCl_2_ 100 mM, (**h**) emulsion containing CaCl_2_ 100 mM.

**Figure 7:**
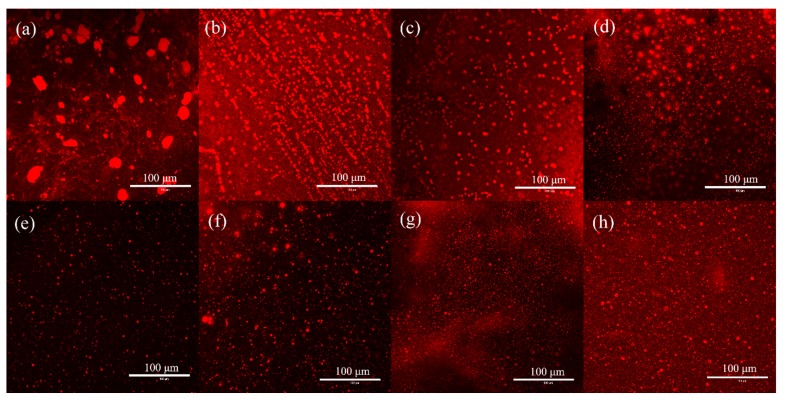
The microstructures of emulsions (**a**) without the XEHR, and at pH of (**b**) 6.86, (**c**) 4, and (**d**) 10, and with (**e**) 100 mM NaCl, (**f**) 100 mM KCl, (**g**) 100 mM MgCl_2_, and (**h**) 100 mM CaCl_2_. The scale bars indicate 100 μm.

The particle sizes and optical micrographs of emulsion samples made under different conditions are provided in Figure 6 and Figure 7, respectively. The emulsion without the XEHR was evidently uneven and unstable and showed large, irregular droplets with sizes between 10 and 20 µm. After adding the XEHR, the droplet sizes decreased to 6 to 10 µm under acidic conditions, and these droplets were relatively uniform in size and shape. Under alkaline and neutral conditions, smaller droplets (2 to 4 µm) appeared in the emulsions. The average droplet size in the 100 mM MgCl_2_ emulsion was the smallest (3 to 7 µm), and the droplet morphology was more uniform. The droplet sizes were 1.5 to 3 µm and 6 to 10 µm in the emulsion containing CaCl_2_, indicating both large and small droplets. The particle sizes in the emulsion containing NaCl were between 4 and 10 µm, with few variations in shape. The particle sizes in the KCl emulsion were the largest among the various salt emulsions (5 to 10 µm), with the majority of droplets having a size of approximately 8 µm. The effect of salt on the droplet size is attributed to the effect of charge screening [48] and cationic gelling agent [42]. The larger the viscosity of the continuous phase in the emulsion, the smaller the droplet agglomeration tendency, and the smaller the particle size, the more evenly dispersed it is in the emulsion network structure [45], which means that the stability of the emulsion is stronger. This can also be confirmed by comparing the experimental results of emulsion size and emulsion stability.

Zeta potentials also provide useful information regarding the stability of an emulsion (Table 5). It is reported that a higher zeta potential value is accompanied by a more stable emulsion. Generally, an absolute zeta potential value of 25–30 mV is typically used as an approximate threshold for stability [49]. The zeta potential of the emulsion without the XEHR was less than 20 mV, indicating an unstable mixture. The zeta potentials of the acidic emulsions were also found to be positive and small. It can be seen that in neutral and saline environments, the surface charge of the droplet was negative. Only when pH = 4 was applied did the Zeta potential become positive and the absolute value decrease. This proves that the acidic environment will neutralize and change the negative charge on the surface of the droplet, which may be the reason why the emulsion was slightly less stable under acidic conditions. The zeta potentials of the emulsions containing salts were higher than those without salts at all pH values. The highest zeta potential was obtained using MgCl_2_ (−41.75 mV), indicating that this was the most stable emulsion. The Zeta potential test results coincide very well with the ESI and other test results. Therefore, this experiment has proved the high stability of XEHR salt-containing emulsion from many aspects and its good emulsifying properties under various environmental conditions.

#### 2.3.4. Emulsion Rheology

**Figure 8:**
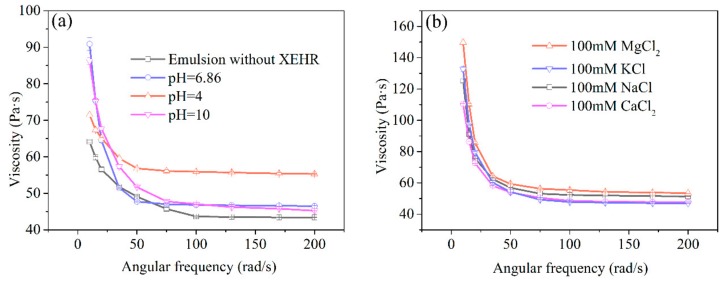
The viscosities of emulsions (**a**) without the XEHR and at different pH (**b**) containing various salts.

To better understand the relationship between emulsion structure and macroscopically measurable properties, rheology tests were carried out to ascertain the relationship between the internal structure and flow characteristics. The apparent viscosity of an emulsion plays a key role in the formation and stability of the mixture. Specifically, increases in viscosity inhibit the free movement of the two phases as well as phase separation and flocculation. The viscosity changes of the emulsions at various shear rates and with different pH environments and salt types are presented in Figure 8. After XEHR addition, the viscosity of the emulsion decreased sharply with increases in shear rate, thus showing shear thinning behavior. The viscosity of emulsion was obviously the lowest when XEHR was not added. After adding XEHR, the viscosity of the emulsion increased in all pH environments. The appearance of non-Newtonian shear thinning was most evident in the case of the mixtures incorporating salts, which showed higher viscosities in the range of 20 to 70 Pa·s. This result is in agreement with the increased ESI values of the salt-based emulsions (Table 4). These data suggest that these emulsions had stronger oil droplet networks with smaller average droplet sizes. Studies have shown that the addition of substances that are not adsorbed in the emulsion can increase the viscosity of the aqueous phase and limit the movement of droplets, thus improving the stability of the emulsion [50].

## 3. Materials and Methods

### 3.1. Materials

Hydrogenated rosin (N grade, composed of approximately 95% colophony acids, acid value 191.79 mg KOH/g) was acquired from Guangxi Wuzhou Pine Chemicals (Guangxi, China). First-class soybean oil was purchased in a local supermarket. Rhodamine B was obtained from the Damao Chemical Reagent Factory (Tianjin, China). Food-Grade xylitol was obtained from the Futaste Pharmaceutical Co., Ltd. (Shandong, China). Carbon dioxide (99.9%) was obtained from the Nanning Air Separation Gas Co., Ltd. (Guangxi, China).

### 3.2. Preparation and Purification of XEHR

#### 3.2.1. Use High-Pressure CO_2_ as Catalyst

The esterification reaction between the hydrogenated rosin and xylitol was catalyzed by high-pressure CO_2_ in an FYX-2 (Jingyi reaction kettle Co., Ltd., Dalian, China) stainless steel autoclave. The hydrogenated rosin and xylitol were added to the autoclave in the desired proportion, after which the unit was sealed and air was removed using a vacuum pump. After the seal was tested, the reactor was filled with CO_2_ and evacuated again three times. The reaction was initiated by injecting a specific quantity of CO_2_ as a gaseous catalyst and heating the reactor. During the reaction process, the exhaust valve was opened once an hour to discharge water vapor and allow the reactor to be refilled with CO_2_.

Following the reaction, trace unreacted xylitol was possibly retained in the XEHR, which would be expected to induce errors in acid values for the products. To eliminate these effects, 50 g of the XEHR product was mixed with 80 g turpentine and 100 g saturated brine after the reaction, followed by stirring for 30 min in a 70 °C water bath. The turpentine phase was subsequently separated and washed three times with distilled water. Finally, the turpentine and residual water were removed by distillation under vacuum to give the final product.

#### 3.2.2. Use ZnO as Catalyst

Hydrogenated rosin and xylitol were added to the autoclave according to the required proportion, 0.8% ZnO was added (based on the quality of hydrogenated rosin), and then the device was sealed and the reactor was heated to start the reaction. During the reaction process, the exhaust valve was opened once an hour to discharge water vapor.

The purification steps after the reaction are the same as those in Section 3.2.1.

#### 3.2.3. Without Using Any Catalysts

Hydrogenated rosin and xylitol were added to the autoclave according to the required proportion, and then the device was sealed and the reactor was heated to start the reaction. During the reaction process, the exhaust valve was opened once an hour to discharge water vapor.

The purification steps after the reaction are the same as those in Section 3.2.1.

### 3.3. Fundamental Properties of XEHR

#### 3.3.1. Determination of Conversion Values

The acid value was the main parameter used to ascertain the quality of the XEHR, and acid values were converted using the equation:

(1) $$\mathrm{conversion}(\%)=(1-\frac{N_{E}}{N_{R}})*100\%$$

where *NR* and *NE* are the acid values of the original hydrogenated rosin and of the XEHR, respectively, both of which could be determined based on the ASTM D-465 standard [51].

#### 3.3.2. HLB Values

Hydrophilic–lipophilic balance (HLB) values were determined by accurately weighing 1 g portions of the XEHR samples into 125 mL Erlenmeyer flasks and adding 30 mL of an isopropanol/ benzene (100:15 *v*/*v*) mixture to dissolve the samples. Following this, each sample was titrated with double-distilled water until persistent turbidity was obtained. In the method reported by Greenwald et al. [52], the volume of water added is termed the water index. A calibration curve was constructed using span-80 (HLB = 4.3) and tween-80 (HLB = 15.0) mixtures, and intermediate HLB values were calculated using the equation:

(2) $${\mathrm{HLB}}_{mix}=\frac{w_{A\;}HLB_{A}+w_{B\;}HLB_{B}}{w_{A\;}+w_{B}},\;w_{A\;}+w_{B}=1g$$

where $HLB_{A}$ and $HLB_{B}$ are the HLB values obtained for the span-80 and tween-80.

#### 3.3.3. ICP-AES

The original hydrogenated rosin, XEHR prepared with high-pressure CO_2_, and XEHR prepared with ZnO as a catalyst under the same conditions were analyzed by inductively coupled plasma-atomic emission spectroscopy (ICP-AES) to determine the mass fractions of Cu, Fe, Zn, Pb, and Ni in these materials.

### 3.4. Characterization of XEHR

#### 3.4.1. Fourier Transform Infrared Spectroscopy

Prior to analyses by a Shimadzu Iraffinity-1s Fourier transform infrared (FTIR) spectroscopy (Shimadzu, kyoto, Japan), samples of the hydrogenated rosin or XEHR were mixed with KBr powder and pressed into transparent sheets. The main functional groups in these samples were analyzed over the wavenumber range of 4000–400 cm^−1^.

#### 3.4.2. NMR

Analyses by ^1^H and ^13^C nuclear magnetic resonance (NMR) spectroscopy were performed with a Bruker ADVANCE III HD600 spectrometer (Bruker, Switzerland). A 40 mg portion of each sample was dissolved in 600 mL CDCl_3_, and spectra were acquired at 298 K. All chemical shifts are reported relative to that of tetramethylsilane (TMS).

#### 3.4.3. GPC

Gel permeation chromatography (GPC) analyses were conducted using a Waters 1525 instrument with a refractive index (RI) detector and a Phenogel column (00H-0441-K0). Mobile phase was THF. The samples were diluted in THF at a concentration of 5 mg/mL, and a 1.00 mL/min flow rate was employed together with a column temperature of 40 °C.

### 3.5. Emulsion Preparation

#### 3.5.1. Surface and Interfacial Tensions

The equilibrium surface tension was determined using the ring method (DCAT21 tensiometer), while the interfacial tension was measured with a JJ2000B spinning droplet tensiometer (POWEREACH, Shanghai, China). During these trials, the vessel containing the solution to be measured was immersed in a constant-temperature water bath held at 25.0 ± 0.05 °C. The tensiometer was calibrated with double-distilled water. The deviation between replicate surface tension measurements was less than 0.2 mN/m.

#### 3.5.2. Emulsification Properties

In emulsification tests, the XEHR was dissolved in soybean oil to form a 2% (*w*/*v*) solution, after which a salt emulsion was prepared by mixing aqueous NaCl, KCl, CaCl_2_, or MgCl_2_ solutions with the soybean oil solution containing the 2% XEHR so as to obtain a water-to-oil volume ratio of 1:4. Varying pH values of 4, 6.86, or 10 were prepared by adding HCl or NaOH to these mixtures. (Such as for XEHR water in oil emulsion containing 10 mM NaCl, the final 100 mL emulsion contained 80 mL soybean oil, 20 mL water, 1 mmol NaCl, and 1.6 g XEHR). These mixtures were homogenized twice using an AH-100D high-pressure homogenizer (ATS Engineering, Ltd., Toronto, ON, Canada) at a pressure of 18,000 bar to form water/oil emulsions. The emulsifying activity index (EAI) and emulsifying stability index (ESI) of the emulsions containing the XEHR were determined by the improved turbidimetric method. In this method, aliquots of each emulsion (100 μL) were taken from the bottom of the bottle both immediately after preparation of the emulsion and after 10 min, then diluted with 5 mL of a 0.1% sodium dodecyl sulfate (SDS) solution [53]. An ultraviolet/visible (UV/vis) spectrophotometer (S2000) was used to determine the absorbance of each diluted emulsion at 500 nm. The EAI and ESI were calculated using the equations:

(3) $$\mathrm{EAI}\;\left(\frac{m^{2}}{g}\right)=2T\;\left(A_{0}\cdot \frac{\mathrm{dilution}}{C\cdot \theta \cdot 10000}\right)$$

(4) $$\mathrm{ESI}\;\left(\%\right)=\frac{A_{10}}{A_{O}}*100$$

where *T* is 2.303, *A_0_* is the absorbance at 500 nm measured immediately after homogenization, “dilution” is 100, *C* is the concentration of XEHR (g/mL) in the oil phase prior to emulsion formation, *θ* is the oil volume fraction in the emulsion, and *A_10_* is the absorbance at 500 nm after 10 min [46,54].

#### 3.5.3. Droplet Size Distribution and Zeta Potentials

A Zetasizer Nano ZS laser nanoparticle size analyzer (Malvern Co., Worcestershire, UK) was used to measure the particle sizes and surface charges in the emulsions at 25 °C. Prior to these measurements, each emulsion was diluted by a factor of 100 using soybean oil. The hydrodynamic diameter and zeta potential of the particles provided herein represent the averages of at least three measurements [11].

#### 3.5.4. Confocal Laser Scanning Microscopy

The microstructures of the freshly prepared emulsions were analyzed by confocal laser scanning microscopy (CLSM; Olympus, FV3000, Tokyo, Japan). For this purpose, rhodamine B was added to the emulsion according to the ratio of 10 µg/mL, and a 0.2 mL aliquot of the emulsion was obtained and diluted with 1.8 mL of soybean oil. A small drop of the diluted emulsion was transferred to a glass slide, covered with a slip, and then immediately observed. A 40 × objective lens was used for the surface observations of the emulsion droplets.

#### 3.5.5. Rheological Behaviors

Flow curves were recorded for emulsion samples at 25 °C shear rates in the range of 1–100 s^−1^, using an NDJ-1F Brookfield rotational viscometer (Shanghai, China) for the determination of viscosity. A concentric cylinder geometry (rotor 21) was used for these measurements.

## 4. Conclusions

The synthesis of XEHR using a “green” high-pressure CO_2_ system was demonstrated with a 91.51% yield. The product did not contain heavy metal residues. Employing a soybean oil/water volume ratio of 4:1, the XEHR was used to prepare emulsions via high-pressure homogenization. This ester exhibited satisfactory interfacial activity and emulsification performance in both acidic and alkaline environments and in the presence of various salts. The microstructures of the emulsions showed that the presence of salts improved droplet dispersion to generate more stable mixtures. This effect may be related to the adsorption of ions on the surfaces of the emulsion droplets and to the type of ion. This work provides data regarding the physicochemical and emulsifying properties of a new bio-based emulsifier, and it suggests that XEHR may have numerous applications.

## Figures and Tables

**Table 1:** Esterification conversion values under different catalytic conditions. Reaction conditions: (1) no catalyst, (2) the traditional ZnO catalyst (0.8%), (3) high-pressure CO_2_ as the catalyst (4 MPa). Common reaction conditions: temperature = 250 °C, time = 4 h, molar ratio of hydrogenated rosin to xylitol = 1:1.

Samples	Conversion (%)
**XEHR** (without catalyst)	63.65 ± 0.05 ^c^
**XEHR** (ZnO as catalyst)	78.52 ± 0.06 ^b^
**XEHR** (high-pressure CO_2_ as catalyst)	91.54 ± 0.03 ^a^

Values are given as the mean ± SD from triplicate determinations; ^a–c^ indicates that different letters in the same column differ significantly (*p* < 0.05); XEHR, xylitol ester of hydrogenated rosin.

**Table 2:** Cu, Fe, Pb, Zn, and Ni concentrations in the hydrogenated rosin (row 1), XEHR generated by ZnO catalysis (row 2), and XEHR generated by high-pressure CO_2_ catalysis (row 3) as determined by ICP-AES.

Samples	Cu (%)	Fe (%)	Ni (%)	Pb (%)	Zn (%)
Hydrogenated rosin	<0.0004	<0.0004	<0.0004	<0.0004	0.0013
XEHR (ZnO as catalyst)	<0.0004	<0.0004	<0.0004	<0.0004	0.20
XEHR (high-pressure CO_2_ as catalyst)	<0.0004	<0.0004	<0.0004	<0.0004	0.0018

**Table 3:** GPC data for the hydrogenated rosin and XEHR.

Retention Time (min)	Mn	Mw	Polydispersion Index
10.184	276	319	1.15367
9.618	772	844	1.09319
9.887	427	433	1.01454
10.178	266	271	1.01699

**Table 4:** Emulsifying activity index (EAI) and emulsifying stability index (ESI) values for emulsions made with the XEHR with different pH, salt concentrations, and salt species.

Samples	Emulsifying Activity Index (m^2^/g)	Emulsifying Stability Index (%)
Emulsion without XEHR	4.60 ± 0.31 ^g^	70.0 ± 0.64 ^g^
pH = 6.86	7.94 ± 0.09 ^c^	84.69 ± 0.38 ^bc^
pH = 4	7.11 ± 0.11 ^f^	75.56 ± 1.57 ^ef^
pH = 10	7.92 ± 0.18 ^cd^	82.65 ± 1.28 ^cd^
10 mM NaCl	7.33 ± 0.35 ^ef^	72.71 ± 0.70 ^f^
50 mM NaCl	7.64 ± 0.39 ^de^	78.56 ± 1.75 ^de^
100 mM NaCl	9.33 ± 0.20 ^a^	84.36 ± 0.29 ^bc^
100 mM KCl	8.37 ± 0.33 ^bc^	89.26 ± 3.08 ^ab^
100 mM MgCl_2_	9.52 ± 0.13 ^a^	94.53 ± 1.53 ^a^
100 mM CaCl_2_	8.75 ± 0.13 ^b^	80.20 ± 1.12 ^cd^

Values are given as the mean ± SD from triplicate determinations; ^a–g^ indicates that different letters in the same column differ significantly (*p* < 0.05).

**Table 5:** Zeta potentials of emulsion samples under different conditions.

Samples	Zeta Potentials (mV)	Samples	Zeta Potentials (mV)
Emulsion without XEHR	−16.41 ± 3.09 ^c^	100 mM NaCl	−37.58 ± 1.01 ^a^
pH = 6.86	−35.72 ± 2.42 ^d^	100 mM KCl	−39.45 ± 5.36 ^d^
pH = 4	27.88 ± 1.41 ^b^	100 mM MgCl_2_	−41.75 ± 4.72 ^d^
pH = 10	−37.51 ± 5.29 ^d^	100 mM CaCl_2_	−32.61 ± 3.26 ^ab^

Values are given as the mean ± SD from triplicate determinations; ^a–d^ indicates that different letters in the same column differ significantly (*p* < 0.05).

## Supplementary Materials

The following are available online at https://www.mdpi.com/1420-3049/25/2/302/s1, Figure S1: FT-IR spectra of the XEHR and the hydrogenated rosin; Figure S2: ^1^H and ^13^C NMR spectra obtained from the XEHR.
