# Supplementary material for: The Emulsifying Properties of Hydrogenated Rosin Xylitol Ester as a Biomass Surfactant for Food: Effect of pH and Salts
Source: Molecules. 2020 Jan 12;25(2):302. doi: 10.3390/molecules25020302 (PMC7024234; doi:10.3390/molecules25020302)
Supplement: Supplementary file 1 [file molecules-25-00302-s001.pdf]

Supplementary Materials

The emulsifying properties of hydrogenated rosin xylitol ester as a biomass surfactant for food: effect of pH and salts

Hong Qiu[1], Xiaopeng Chen[1,2], Xiaojie Wei[1,2], Jiezhen Liang[1,2], Dan Zhou[1], Linlin Wang[1,2*]

[1]School of Chemistry and Chemical Engineering, Guangxi University, Nanning 530004, People's Republic of China

[2]Guangxi Key Laboratory of Petrochemical Resources Processing and Process Intensification Technology, Guangxi University, Nanning 53004, People's Republic of China

* Correspondence: wanglinlin1971@sina.com; Tel.: +86-771-3272702

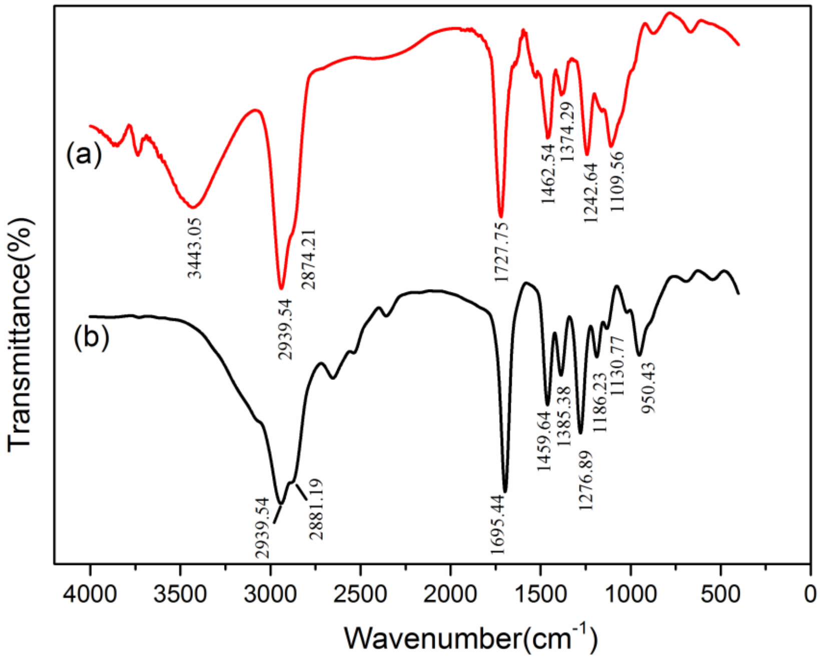

Fig. S1: FT-IR spectra of (a) the XEHR and (b) the hydrogenated rosin.

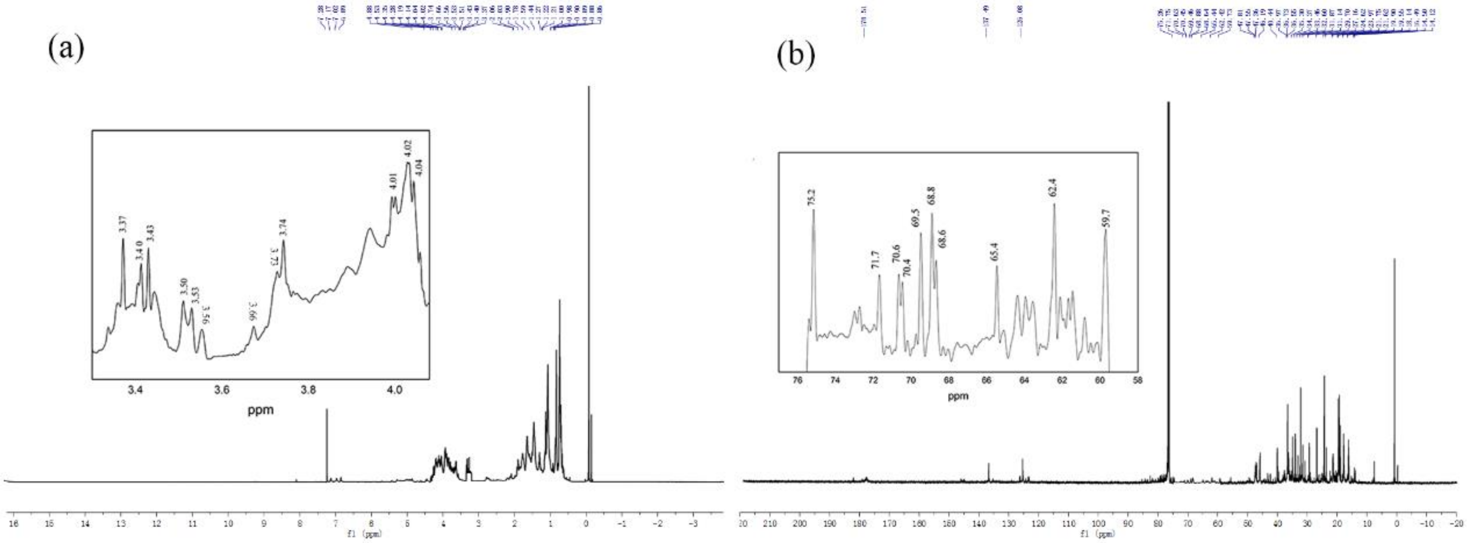

Fig. S2: (a) ^{1}H and (b) ^{13}C NMR spectra obtained from the XEHR.
